# Mitochondrial Fragmentation and Long Noncoding RNA *MALAT1* in Diabetic Retinopathy

**DOI:** 10.3390/ijms26136429

**Published:** 2025-07-03

**Authors:** Renu A. Kowluru, Jay Kumar

**Affiliations:** Ophthalmology, Visual and Anatomical Sciences, Kresge Eye Institute, Wayne State University, Detroit, MI 48201, USA

**Keywords:** diabetic retinopathy, DNA methylation, long noncoding RNA, *MALAT1*, mitochondria

## Abstract

Mitochondria are dynamic in nature and depending on the energy demand they fuse and divide. This fusion-fission process is impaired in diabetic retinopathy and the promoter DNA of *Mfn2*, a fusion gene, is hypermethylated and its expression is downregulated. Long noncoding RNAs (RNAs with >200 nucleotides that do not encode proteins) can regulate gene expression by interacting with DNA, RNA, and proteins. Several LncRNAs are aberrantly expressed in diabetes, and among them, *MALAT1* is upregulated in the retina, altering the expression of the genes associated with inflammation. Our aim was to investigate *MALAT1*’s role in mitochondrial dynamics in diabetic retinopathy. Using *MALAT1*-siRNA-transfected human retinal endothelial cells (HRECs) and human retinal Muller cells (RMCs) incubated in 20 mM D-glucose, Mfn2 expression and activity and its promoter DNA methylation were quantified. Mitochondrial integrity was evaluated by analyzing their fragmentation, ultrastructure, membrane potential, and oxygen consumption rate. Compared to normal glucose, high glucose upregulated *MALAT1* expression and downregulated Mfn2 expression and activity in both HRECs and RMCs. *MALAT1*-siRNA ameliorated the glucose-induced increase in *Mfn2* promoter DNA hypermethylation and its activity. *MALAT1*-siRNA also protected against mitochondrial fragmentation, structural damage, and reductions in the oxygen consumption rate. In conclusion, the upregulation of *MALAT1* in diabetes facilitates *Mfn2* promoter DNA hypermethylation in retinal vascular and nonvascular cells, leading to its suppression and the accumulation of the fragmented/damaged mitochondria. Thus, the regulation of *MALAT1* has the potential to protect mitochondria and provide a possible new target to inhibit/prevent the blinding disease in diabetic patients.

## 1. Introduction

Diabetic retinopathy is one of the most feared complications of diabetes. Uncontrolled hyperglycemia attacks retinal vascular and nonvascular components, ultimately leading to blindness [1,2,3,4]. Many metabolic abnormalities initiated by hyperglycemia have been implicated in the pathogenesis of diabetic retinopathy, but the exact molecular mechanism responsible for its development remains unclear. Among different metabolic abnormalities, oxidative stress–mitochondrial dysfunction is considered to play a central role in the development of diabetic retinopathy [5,6,7,8,9,10].

Mitochondria are highly dynamic organelles, and to maintain their functional integrity, they continuously undergo coordinated cycles of fission and fusion. Mitochondrial dynamics also help in removing damaged components from a mitochondrion, or the entire impaired mitochondria is removed by mitophagy. An imbalanced mitochondrial dynamic disrupts mitochondrial function and is implicated in several diseases, including neurodegenerative disorders, cancers, cardiovascular diseases, and diabetic complications such as diabetic retinopathy [11,12,13,14,15]. Mitochondrial fusion involves the merging of two outer membranes, followed by the joining of inner membranes, and is mediated by the dynamin family GTPases mitofusin 1 and 2 (Mfn1 and Mfn2) and optic atrophy 1. Their fission begins with the division of the inner mitochondrial membrane, followed by the scission of the outer mitochondrial membrane by the GTPase Drp1 [16,17]. In diabetic retinopathy, mitochondrial dynamics are impaired with decreased fusion and increased fission; while Mfn2 expression and its GTPase activity are downregulated, Drp1 is upregulated and activated [14,18,19,20]. Previous studies have shown that epigenetic modifications, especially the hypermethylation of *Mfn2* promoter DNA, play a major role in suppressing its gene transcripts [14,21]; however, the mechanism of *Mfn2* promoter hypermethylation is unclear.

Long non-coding RNAs (LncRNAs), the non-protein-coding transcripts that are longer than 200 nucleotides, can regulate gene expression at the epigenetic, transcriptional, and post-transcriptional levels, and they affect many biological and pathological processes [22,23,24]. Several LncRNAs are aberrantly expressed in diabetes and are implicated in diabetic complications, including cardiomyopathy and retinopathy [25,26,27,28,29,30,31,32]. LncRNAs can recruit the enzymes intimately associated with the methylation of cytosine (DNA methyl transferases, Dnmts) or interact with histone-modifying enzymes, regulating the expression of the target gene [33,34,35,36]. Among the aberrantly expressed LncRNAs in diabetes, metastasis-associated lung adenocarcinoma transcript 1 (*MALAT1*), a highly conserved LncRNA [37], is upregulated in diabetes, and it is shown to regulate oxidative stress–mitochondrial dysfunction [29,30,38]. *MALAT1* can also act as an epigenetic regulator by facilitating DNA methylation and histone modifications [35,38,39]. However, it is unclear if *MALAT1* has any role in altering the methylation status of *Mfn2* promoter DNA, suppressing its expression.

The aim of this study is to investigate the role of *MALAT1* in the regulation of Mfn2, especially focusing on its promoter DNA methylation. Using both vascular (human retinal endothelial cells, HRECs) and nonvascular (human retinal Muller cells, HMCs) components of the retina, the effect of *MALAT1* regulation on *Mfn2* promoter DNA methylation and mitochondrial structure–function is investigated.

## 2. Results

**Figure 1:**
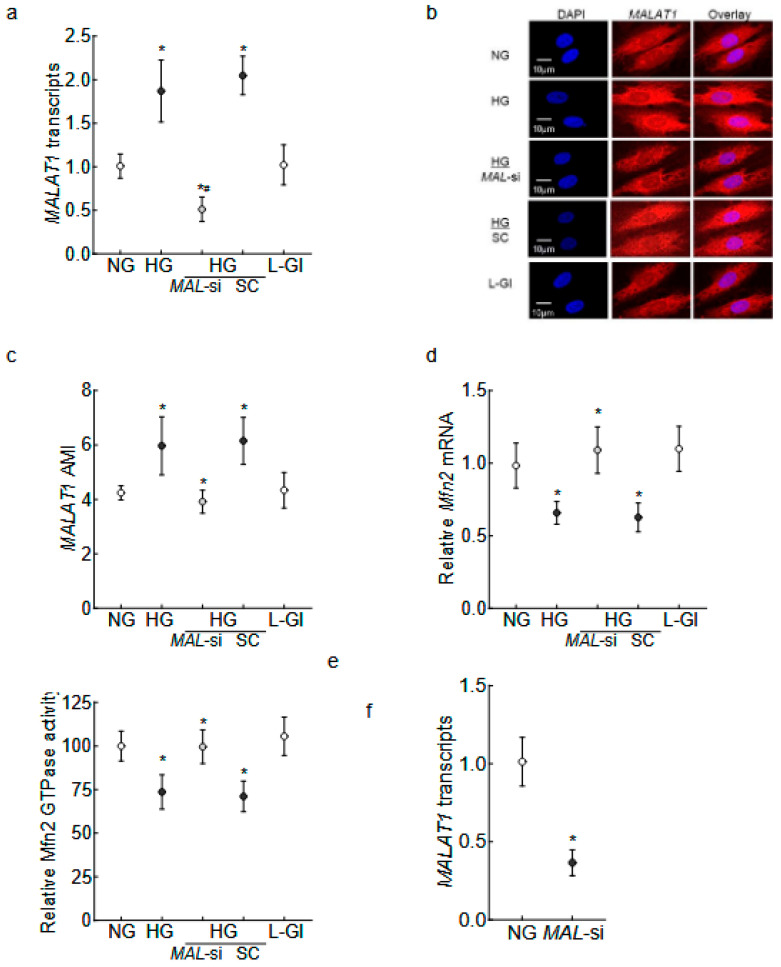
Effect of *MALAT1*-siRNA on the glucose-induced downregulation/inhibition of Mfn2. HRECs incubated in high glucose for 96 h were analyzed for *MALAT1* (**a**) transcripts by qRT-PCR using β-actin as a housekeeping gene and (**b**) expression by RNA FISH. (**c**) AMI of *MALAT1*, calculated from 25–30 cells in each group. Mfn2 (**d**) gene transcripts were quantified by qRT-PCR by employing β-actin as a housekeeping gene, and (**e**) GTPase activity was examined by quantifying phosphate released at 620 nm. Using values from cells in normal glucose as 100%, the percentage change in the phosphate released was calculated. (**f**) Transfection efficiency of *MALAT1*-siRNA. The values in the graphs are represented as mean ± SD, obtained from 3–4 cell preparations, with each measurement made in duplicate/triplicate. NG and HG = 5 mM or 20 mM D-glucose; HG/*MAL*-si and HG/SC = *MALAT1*-siRNA or scrambled control RNA-transfected HRECs in HG, L-Gl = 20 mM L-glucose. * *p* < 0.05 compared with NG and # *p* < 0.05 compared with HG.

Human Retinal Endothelial Cells: As expected [14,29], *MALAT1* transcripts and the Arithmetic Mean Intensity (AMI) of *MALAT1* (obtained from RNA fluorescence in situ hybridization, RNA-FISH) were significantly increased in high glucose (*p* < 0.01); and *Mfn2* gene expression was downregulated by 40%, and its GTPase activity was inhibited by 30% compared to values in normal glucose (Figure 1a–e). In the same cell preparation, the regulation of *MALAT1* by its specific siRNA ameliorated the glucose-induced decrease in Mfn2 gene transcripts and GTPase activity. The values obtained from *MALAT1*-siRNA-transfected cells in high glucose were significantly different from untransfected or scrambled RNA-transfected cells in high glucose. The incubation of cells in 20 mM L-glucose, instead of 20 mM D-glucose, had no effect on Mfn2 expression, GTPase activity, and *MALTA1* transcripts. Figure 1f is included to show the >50% transfection efficiency of *MALTA1*-siRNA in HRECs.

**Figure 2:**
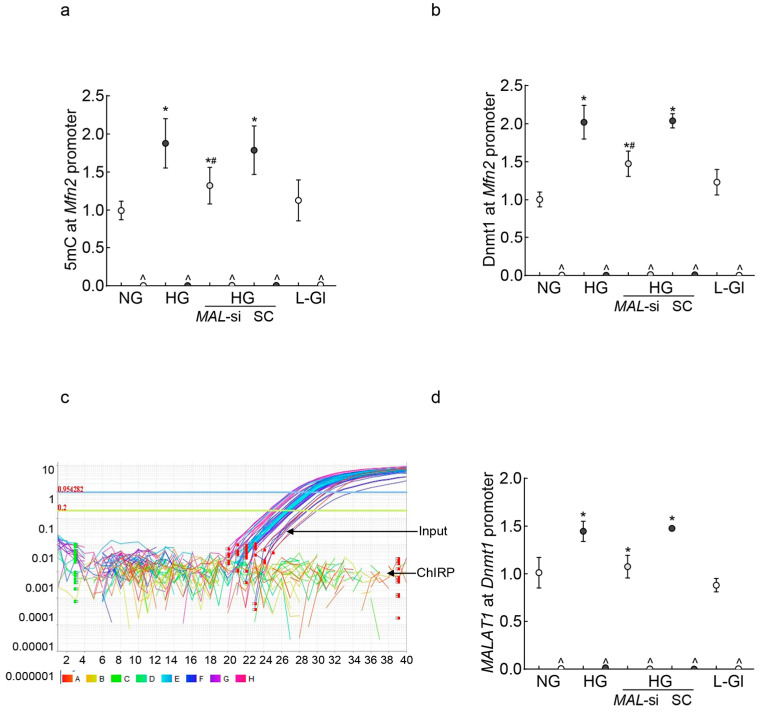
*MALAT1* upregulation and *Mfn2* promoter DNA methylation in HRECs. Using IgG as an antibody control, (**a**) 5mC levels at *Mfn2* promoter DNA were quantified by the methylated DNA capture method, and (**b**) Dnmt1 binding at the *Mfn2* promoter was measured by the ChIP technique. *MALAT1* binding at (**c**) the *Mfn2* promoter was determined by the ChIRP technique, and (**d**) Dnmt1 by the RIP technique. NG and HG = cells in 5 mM or 20 mM D-glucose; HG/*MAL*-si and HG/SC = *MALAT1*-siRNA or scrambled control RNA-transfected cells in HG, L-Gl = 20 mM L-glucose. ^ = IgG antibody control, * *p* < 0.05 vs. NG and # *p* < 0.05 vs. HG.

LncRNAs can regulate gene expression by epigenetic modifications, and *Mfn2* promoter DNA is hypermethylated in diabetes [14,33,35]; the role of *MALAT1* in *Mfn2* DNA methylation was evaluated. As shown in Figure 2a, 5methyl cytosine (5mC) levels at the *Mfn2* promoter were significantly elevated in high glucose compared to normal glucose (*p* < 0.05). The transfection of HRECs with *MALAT1*-siRNA, and not with the scrambled control RNA, attenuated a glucose-induced increase in 5mC levels. Although the values obtained from *MALAT1*-siRNA-transfected cells in high glucose were higher than untransfected cells in normal glucose, they were significantly lower than untransfected cells in high glucose (*p* < 0.05). Consistent with increased 5mC levels, the increased binding of Dnmt1 at the *Mfn2* promoter, as seen in high glucose conditions, was also ameliorated by *MALAT1*-siRNA (Figure 2b). The scrambled control RNA had no effect on glucose-induced increase in either 5mC levels or Dnmt1 binding at the *Mfn2* promoter.

To investigate the mechanism by which *MALAT1* facilitates *Mfn2* promoter DNA hypermethylation, using the Chromatin Isolation by RNA Purification (ChIRP) technique, the binding of *MALAT1* at the *Mfn2* promoter was investigated. Surprisingly, while the input control sample had good amplification, no amplification was observed in the ChIRP sample, suggesting that *MALAT1* might not be directly interacting with the *Mfn2* promoter to methylate its DNA (Figure 2c). However, *MALAT1* can also elevate the DNA methylation status of a gene by recruiting Dnmt1 to its promoter region [40,41]; *MALAT1* binding at the *Dnmt1* promoter was investigated. As shown in Figure 2d, *MALAT1* binding at the *Dnmt1* promoter was significantly increased in high glucose compared to normal glucose, and the inhibition of *MALAT1* by its siRNA prevented a glucose-induced increase in *MALAT1–Dnmt1* interactions. The values obtained from untransfected cells in normal glucose, or 20 mM L-glucose, were not different from those of *MALAT1*-siRNA-transfected cells in high glucose.

**Figure 3:**
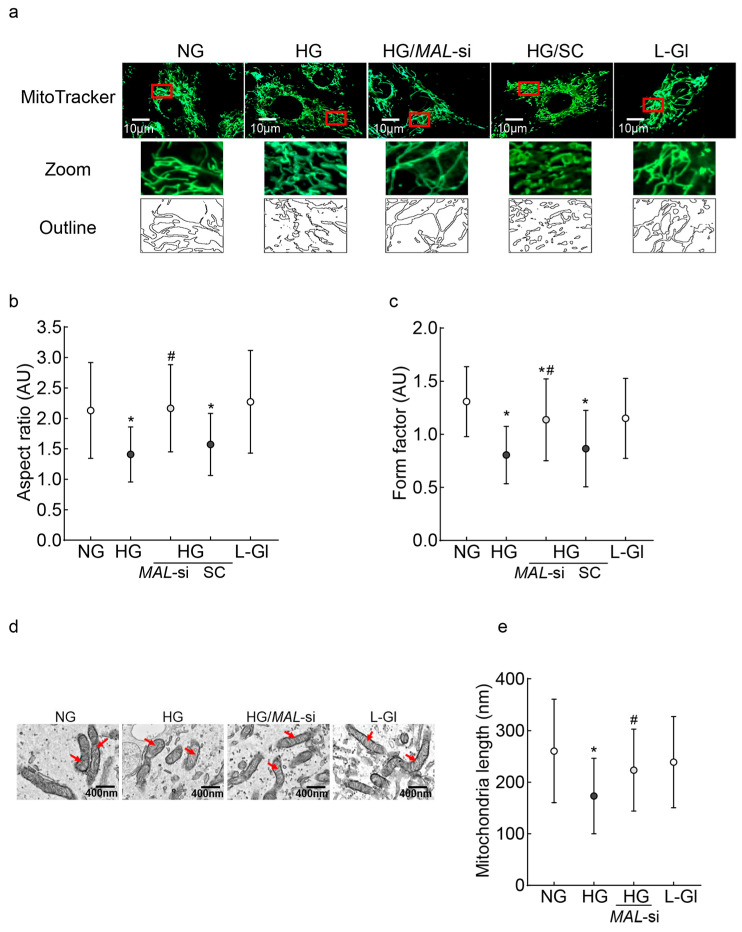
Mitochondrial fragmentation and ultrastructure: (**a**) Mitochondrial fragmentation was evaluated by live cell imaging using an ApoTome fluorescence microscope and a 63X objective in HRECs stained with MitoTracker green. The red box area is zoomed, and the outlines are plotted using ImageJ software, version 1.53K. (**b**,**c**) Aspect ratio and form factor were calculated by ImageJ software in fluorescent images converted to 8-bit images. Values are mean ± SD obtained from 10 to 12 images/conditions with 3–4 cells/image. (**d**) Representative electron micrographs of cells; red arrows point to the mitochondria; (**e**) mitochondrial length was measured using ImageJ software, and the values are represented as mean ± SD from 10 to 12 images/experimental conditions with 8–10 mitochondria/image. NG and HG = 5 mM or 20 mM D-glucose; HG/*MAL*-si and HG/SC = *MALAT1*-siRNA or scrambled RNA-transfected cells in HG, L-Gl = 20 mM L-glucose.* *p* < 0.05 vs. NG and # *p* < 0.05 vs. HG.

Since the downregulation of *Mfn2* results in impaired mitochondrial fusion, leading to the accumulation of fragmented mitochondria, the effect of *MALAT1*-siRNA on mitochondrial fragmentation was investigated. As shown in Figure 3a, compared to untransfected cells, *MALAT1*-siRNA-transfected cells had less fragmentation in high glucose. Similarly, measurements of the aspect ratio (ratio between the major and minor axes of each mitochondrial object) and the form factor, which represents a combined evaluation of the length and degree of the branching of the mitochondrial network [42,43], showed significantly higher values in *MALAT1*-siRNA-transfected cells in high glucose, compared to the unatranfected cells in high glucose (Figure 3b,c). As fragmented mitochondria are structurally and functionally unstable, we investigated the effect of *Mfn2* downregulation on mitochondrial ultrastructure. Mitochondria in high glucose were swollen, and their cristae were fragmented; however, mitochondria from *MALAT1*-siRNA-transfected cells in high glucose were elongated and had regular cristae structure, as seen in untransfected cells in normal glucose or 20 mM L-glucose (Figure 3b). Furthermore, compared to cells in normal glucose, mitochondrial length was significantly reduced in high glucose (*p* < 0.05); however, mitochondrial length of *MALAT1*-siRNA-transfected cells in high glucose and untransfected cells in normal glucose was similar (*p* > 0.05; Figure 3d,e).

**Figure 4:**
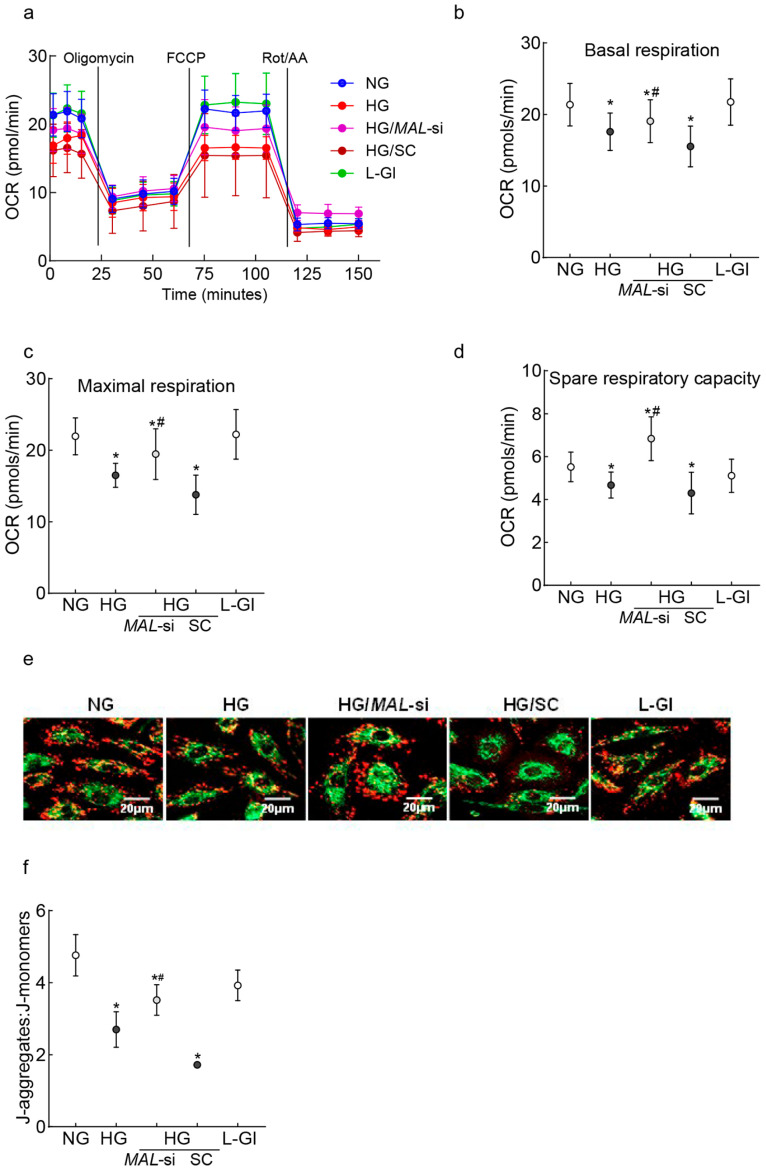
Effect of the protection of *Mfn2* downregulation on mitochondrial damage. (**a**) Oxygen consumption was measured by a Seahorse XF Analyzer Cell Mito Stress Test Kit. Each measurement was repeated 3–5 times using four or more wells/group, and the values are presented as mean ± SD from 3 to 4 HREC preparations. (**b**–**d**) represent basal respiration, maximal respiration, and spare respiratory capacity, respectively. (**e**) Representative image showing retinal endothelial cells stained with JC-1, and (**f**) the ratio of J-aggregates (red) and J-monomers (green). NG and HG = cells in 5 mM or 20 mM D-glucose; HG/*MAL*-si and HG/SC = *MALAT1*-siRNA or scrambled control RNA-transfected cells in HG, L-Gl = 20 mM L-glucose.* *p* < 0.05 vs. NG and # *p* < 0.05 vs. HG.

Mitochondrial cristae contain protein complexes responsible for oxidative phosphorylation, and damaged cristae result in poor respiration rates [44]. The effect of *MALAT1*-*Mfn2* on mitochondrial oxidation rates was evaluated. The oxygen consumption rate (OCR) in high-glucose-incubated cells was significantly lower vs. cells in normal glucose; *MALAT1*-siRNA, but not the control scrambled RNA, ameliorated glucose-induced decreases in mitochondrial respiration (Figure 4a). The amelioration of the glucose-induced decrease in respiration rates was further confirmed by significantly higher basal and maximal respiration rates and spare respiration capacity in *MALAT1*-siRNA-transfected cells in high glucose compared to untransfected cells in high glucose (Figure 4b,c). In accordance with respiration rate, *MALAT1*-siRNA also protected impairments in mitochondrial membrane potential; although, compared to untransfected cells in normal glucose, the ratio of aggregates to monomers was lower in *MALAT1*-siRNA-transfected cells in high glucose, this ratio was significantly higher compared to untransfected cells in high glucose (*p* < 0.05; Figure 4e,f).

**Figure 5:**
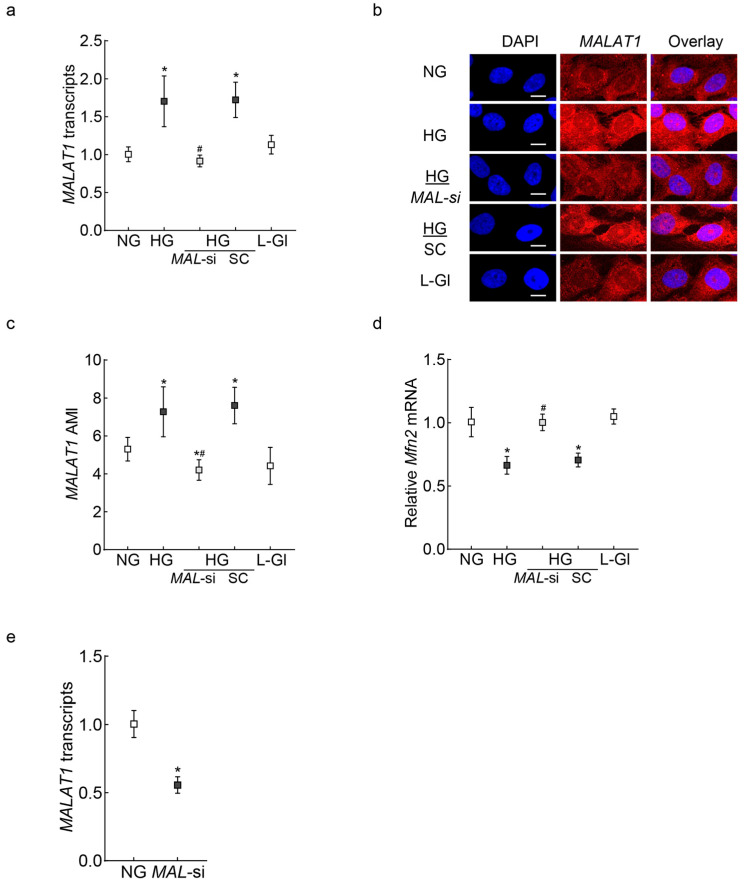
Effect of *MALAT1* regulation on *Mfn2* in retinal Muller cells. RMCs, incubated in high glucose for 96 hrs, were analyzed for *MALAT1* (**a**) transcripts by qRT-PCR using β-actin as a housekeeping gene, and (**b**) expression was analyzed by RNA-FISH. (**c**) Mean AMI of *MALAT1*, calculated from >20 cells/coverslip. (**d**) *Mfn2* gene transcripts, quantified by qRT-PCR, with β-actin as a housekeeping gene. (**e**) Transfection efficiency of *MALAT1*. The values mean ± SD from 3 cell preparations. NG and HG = 5 mM and 20 mM D-glucose, respectively; HG/*MAL*-si and HG/SC = *MALAT1*-siRNA or scrambled RNA-transfected cells in HG; L-Gl = 20 mM L-glucose * *p* < 0.05 vs. NG and # *p* < 0.05 vs. HG.

Human Retinal Muller Cells: Consistent with retinal endothelial cells, in HMCs, *MALAT1* transcripts were upregulated by >1.5 fold, and the AMI of *MALAT*1 (RNA-FISH) was significantly increased in high glucose compared to normal glucose. In the same cell preparation, *Mfn2* transcripts were downregulated by ~40% (Figure 5a–d). The high-glucose-induced decrease in *Mfn2* was significantly ameliorated by *MALAT1*-siRNA, and *Mfn2* gene transcripts in untransfected cells in normal glucose and *MALAT1*-siRNA-transfected cells in high glucose were not different from each other (*p* > 0.05). However, the values of the untransfected cells or scrambled control RNA-transfected cells in high glucose were comparable (*p* > 0.05). Figure 5e shows the ~50% transfection efficiency of *MALTA1*-siRNA in HMCs.

**Figure 6:**
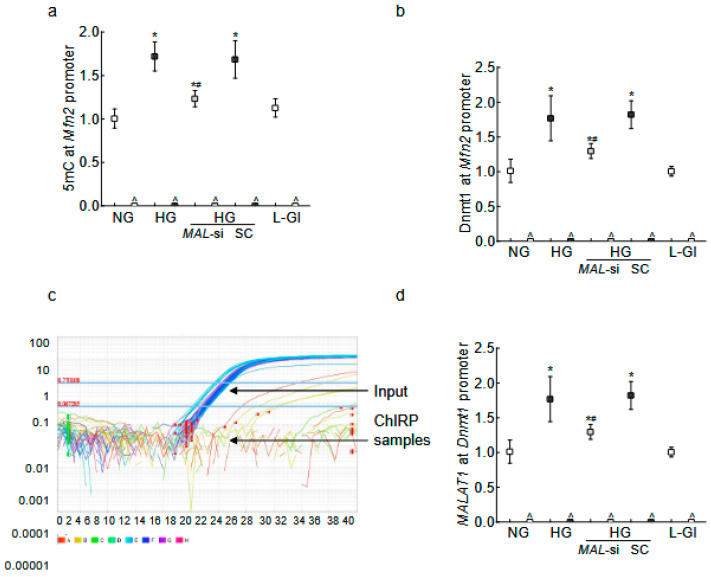
*MALAT1* upregulation and *Mfn2* promoter DNA methylation in RMCs. (**a**) The 5mC levels at the *Mfn2* promoter DNA were quantified by the Methylated DNA capture technique, and (**b**) Dnmt1 binding at *Mfn2* was quantified by the ChIP technique. *MALAT1* binding at (**c**) the *Mfn2* promoter was determined by the ChIRP technique, and (**d**) Dnmt1 by the RIP technique. NG = 5 mM D-glucose; HG = 20 mM D-glucose; HG/*MAL*-si and HG/SC = *MALAT1*-siRNA or scrambled RNA-transfected cells in HG, L-Gl = 20 mM L-glucose. ^ = IgG antibody control; * *p* < 0.05 compared to NG and # *p* < 0.05 compared to HG.

HMCs in high glucose, compared to normal glucose, had elevated 5mC levels and increased Dnmt1 binding at the *Mfn2* promoter, and *MALAT1*-siRNA prevented these increases (Figure 6a,b). Furthermore, consistent with the results from HRECs, although *MALAT1* showed no direct binding with *Mfn2*, its binding with Dnmt1 was upregulated by high glucose (*p* < 0.05 vs. HG), and *MALAT1*-siRNA ameliorated *MALAT1*–Dnmt1 interactions (Figure 6c,d).

**Figure 7:**
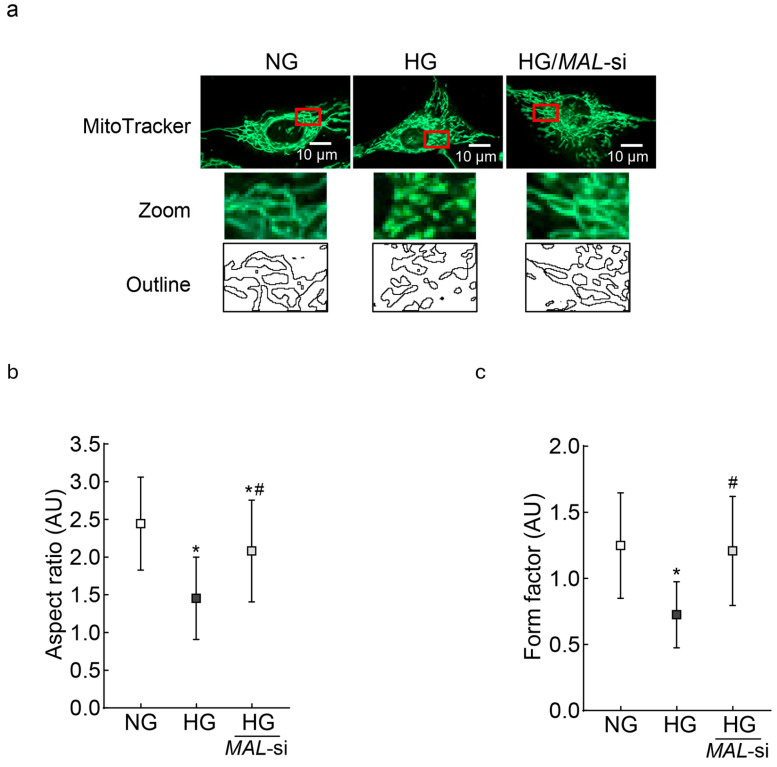
Effect of *MALAT1*-siRNA on mitochondrial fragmentation: (**a**) Using live cell imaging, RMCs stained with MitoTracker green were visualized using an Zeiss ApoTome fluorescence microscope (Carl Zeiss Inc., Baden-Wurttemberg, Germany)and a 63X objective. The zoomed area in the red box was used to plot the outlines. (**b**,**c**) Aspect ratio and form factor were measured by ImageJ software, and the values are mean ± SD from 10 to 12 images/condition with 3–4 cells/image. NG = 5 mM D-glucose; HG and HG/*MAL*-si = untransfected or *MALAT1*-siRNA-transfected cells in 20 mM D-glucose.* *p* < 0.05 vs. NG and # *p* < 0.05 vs. HG.

In the same HMC preparations, high-glucose incubation also resulted in fragmented mitochondria, and the inhibition of *MALAT1* by its siRNA prevented mitochondrial fragmentation (*p* < 0.05; Figure 7a). The aspect ratio and the form factor values were significantly higher in *MALAT1*-siRNA-transfected cells in high glucose, compared to the unatranfected cells in high glucose (Figure 7b,c).

## 3. Discussion

Sustained circulating high glucose damages the metabolically active retina and results in many molecular, functional, and structural abnormalities. The accumulation of ROS and several inflammatory cytokines is increased in the retina, and its vascular and nonvascular cells, including endothelial cells, ganglion cells, and Müller glial cells, are lost. In diabetes, mitochondrial dysfunction is shown to play a central role in the loss of endothelial and Müller cells [5,7,8,9,45]; mitochondrial dynamics, an integral part of mitochondrial homeostasis, is imbalanced, and while their fusion proteins are decreased, the expression of the major fission protein, Drp1, is upregulated, leading to fragmented and dysfunctional mitochondria [14,18,19,20]. Moreover, several LncRNAs are aberrantly expressed in the retina, contributing to the damage of the vascular and nonvascular components of the retina [29,30,38,46]. *MALAT1*, one of the highly conserved LncRNAs, is upregulated in the retina in diabetes, which is shown to increase oxidative stress via regulating the master transcriptional factor Nrf2 and inflammatory mediators. Here, our novel results show that, in both retinal vascular and nonvascular cells, the upregulation of *MALAT1* in diabetes downregulates *Mfn2* by hypermethylating its promoter DNA, and the mechanism for this *MALAT1*-mediated hypermethylation appears to be via increasing *MALAT1*–Dnmt1 interactions, leading to the increased binding of Dnmt1 at the *Mfn2* promoter. The downregulation/inhibition of Mfn2 results in the accumulation of fragmented and dysfunctional mitochondria, and the inhibition of *MALAT1* by its siRNA prevents glucose-induced *Mfn2* promoter DNA hypermethylation and the suppression of its gene and GTPase activity, resulting in healthy mitochondria that are not fragmented and have good oxygen consumption and membrane potential. Retinal vascular and nonvascular components show similar protection by *MALAT1*-siRNA against *Mfn2* promoter DNA hypermethylation, gene suppression, and mitochondrial structural and functional instability. These results strongly suggest that the upregulation of *MALAT1* in diabetes epigenetically modifies *Mfn2* promoter DNA and downregulates its expression, which leads to accumulation of fragmented–dysfunctional mitochondria in both retinal vascular and nonvascular cells.

Long noncoding RNAs, like other noncoding RNAs, were originally considered as ‘transcriptional junk’, but despite some conflicting reports showing all or relatively few, LncRNAs can be functionally active [47,48]. LncRNAs, without coding for proteins, can regulate gene expression by interacting with proteins, DNA, and RNA and/or their combination. They can interact with histone-modifying enzymes that activate or repress gene transcription, act as scaffolds for multiple histone modifiers to regulate histone modification, or recruit DNA methyltransferases to regulate DNA methylation, and their role in epigenetic modifications is now being widely appreciated [33,34,35,36,40]. We have previously shown that, in diabetes, *MALAT1* is upregulated in the retina and its capillary cells, *Mfn2* promoter DNA is hypermethylated, and mitochondrial fission is increased. The data presented here clearly shows that the inhibition of *MALAT1* by its siRNA prevents glucose-induced *Mfn2* promoter DNA hypermethylation and the suppression of its gene and GTPase activity, these results strongly imply the role of *MALAT1* in DNA hypermethylation. In accordance, *MALAT1* is shown to act as a nucleus-to-mitochondria epigenetic messenger in hepatocellular carcinoma cells, and its suppression induces alterations in the CpG methylation of mtDNA and in mitochondrial transcriptomes [49].

As mentioned above LncRNAs can alter gene expressions by binding with proteins, DNA, RNA individually, or with their combination, and *MALAT1* has been shown to alter the histone 3 lysine 27 trimethylation levels of a gene by directly binding with histone methyl transferase Ezh2 in prostate cancer cell lines and HRECs incubated in high glucose [35,38]. Here, our results show that *MALAT1* does not directly interact with the *Mfn2* promoter; instead, it hypermethylates the promoter DNA by recruiting Dnmt1. In support, others have shown that the recruitment of Dnmts by *MALAT1* hypermethylated the DNA of suppressor of cytokine signaling 3 in the Parkinson’s disease model and *CASP3* in the autism model [41,50].

Mitochondrial fusion, the physical merging of the outer and inner mitochondrial membranes of two mitochondria into one, helps the exchange of contents between mitochondria, allowing defective mitochondria to regain essential components of the respiratory chain and mitochondrial DNA [16]. Mitofusins help in the fusion of the outer mitochondrial membrane. Although *Mfn1* and *Mfn2* have some common functions, alterations in *Mfn2* itself can regulate mitochondrial fusion [11]. In diabetes, *Mfn2* is downregulated, and its activity is inhibited, leading to fragmented mitochondria with partial crystolysis and poor respiration [14,15,18,20,51,52,53]. *MALAT1*, a nuclear genome-encoded RNA, is also translocated to the mitochondria [54,55], and its mitochondrial levels are significantly increased in diabetes, resulting in damage to mitochondrial structural and functional integrity [14,20]. The results presented here show that *MALAT1*-siRNA prevents glucose-induced increases in mitochondrial fragmentation and decreases in membrane potential and also preserves their cristae structure and respiration rate, further supporting the role of *MALAT1* in mitochondrial dynamics. Consistent with our results in retinal cells in hyperglycemic milieu, others have shown that in hepatoma HepG2 cells, *MALAT1* functions as an epigenetic factor in the regulation of mitochondrial metabolism, and its binding to mtDNA epigenetically regulates mitochondrial structure and function, including oxidative phosphorylation, mitophagy, and apoptosis [49].

Diabetic retinopathy is conventionally considered a microvascular disease, but sustained hyperglycemia also damages retinal nonvascular cells [4]. Retinal vascular and nonvascular cells, including endothelial cells, pericytes, ganglion cells, and Müller glial cells, are lost in diabetes, and mitochondrial dysfunction is shown to play a major role in the loss of endothelial and Müller cells [6,7,8,45]. Müller cells, one of the most common glial cells, expand across almost the whole width of the retina and envelope retinal capillaries, and their loss in diabetes is associated with the loss of retinal blood barrier integrity and increased vascular permeability [2,56,57]. The results presented here clearly demonstrate that *MALAT1*-mediated DNA methylation plays a significant role in the downregulation of *Mfn2* in diabetes in both endothelial cells and Müller cells, leading to mitochondrial damage/dysfunction, and inhibiting *MALAT1* upregulation by its siRNA protects their mitochondria from undergoing accelerated fission damage.

We acknowledge that our study is focused only on the *MALAT1*-mediated DNA methylation of *Mfn2*; the role of *MALAT1* in modifying histones or acting as an miRNA sponge [58,59,60] to alter *Mfn2* expression remains a possibility. Both mitochondrial fusion-fission and DNA methylation are very dynamic in nature; 5mC formed by DNA methylation can be quickly converted to 5-hydroxy methyl cytosine, leading to gene activation, and we cannot rule out the role of *MALAT1* in altering the DNA methylation status of *Drp1*. Also, the role of *MALAT1* in affecting mitochondrial DNA stability, leading to the development of diabetic retinopathy, cannot be ruled out.

**Figure 8:**
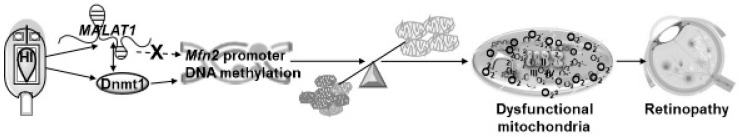
Working model showing the effect of *MALAT1* on the *Mfn2* expression development of diabetic retinopathy.

Mitochondrial fusion–fission, as mentioned above, has a critical role in maintaining mitochondrial integrity, and LncRNAs, though they do not possess a reading frame, have the potential to regulate gene expression via many pathways, including epigenetic modifications. The upregulation of *MALAT1* in diabetes in both vascular and nonvascular cells facilitates the hypermethylation of *Mfn2*, and this results in *Mfn2* gene suppression and the inhibition of its GTPase activity, leading to the accumulation of fragmented/damaged mitochondria (Figure 8). Thus, regulating *MALAT1* could help protect mitochondria and provide a possible new target to inhibit/prevent the development of blinding disease in diabetic patients.

## 4. Methods

Human Retinal Endothelial Cells: HRECs, obtained from Cell Systems Corp (Cat. No. ACBRI 181; Kirkland, WA, USA), were cultured in Dulbecco’s Modified Eagle Medium (DMEM) supplemented with 12% heat-inactivated fetal bovine serum containing 15 μg/mL endothelial cell growth supplement and 1% each of glutamax, insulin, transferrin, selenium, and antibiotic/antimitotic. Confluent cells from the 6th to 8th passage (80–90%) were incubated in a normal glucose (5 mM D-glucose, NG) or high glucose (20 mM D-glucose, HG) incubation medium consisting of DMEM supplemented with 1% heat-inactivated fetal bovine serum, 9% Nu-serum, 1 μg/mL endothelial cell growth supplement, and 1% each of insulin/transferrin/selenium/Glutamax, and antibiotic/antimycotic for 96 h, a duration where mitochondrial dysfunction and cell apoptosis can be observed in these cells [61,62]. HRECs incubated with 20 mM L-glucose (L-Gl), instead of 20 mM D-glucose, were used as an osmotic/metabolic control [29,30].

For transfection with a silencer-select *MALAT1*-siRNA (*MAL*-si; Cat. No. n272231, Invitrogen™, Carlsbad, CA, USA) or scrambled control RNA, the Lipofectamine™ RNAiMAX transfection reagent (Cat. No. 13778075, Invitrogen™, USA) was employed. Transfection efficiency was determined by quantifying the *MALAT1* transcripts using SYBR green-based qRT-PCR [29,30].

Human Retinal Muller Cells: HMCs (Cat no. ABT-TC133L, Accegen Fairfield, NJ, USA) were cultured in an ABM-TM133L culture medium (Accegen), and cells from the 6th to 8th passage were incubated in the incubation medium (DMEM containing 2% FBS, 8% Nu-Serum, and 1% antibiotic/antimycotic) supplemented with normal or high glucose for 96 h. Parallel incubations were run in each experiment, where HMCs were incubated in 20 mM L-glucose instead of 20 mM D-glucose. A group of cells from the 5th to 6th passage was transfected with *MALAT1*-siRNA using Lipofectamine transfection reagent. The purity of HMCs was confirmed by staining with glutamine synthase [63].

Gene Expression: TRIzol-extracted RNA was employed to synthesize cDNA using a High-Capacity cDNA Reverse Transcription kit (Cat. No. 4368814, Applied Biosystems, Waltham, MA, USA). *MALAT1* and *Mfn2* transcripts were quantified by SYBR green-based qRT-PCR, using gene-specific primers and *β-actin* as the housekeeping gene. The primer sequences are listed in Table 1.

RNA Fluorescence In Situ Hybridization: *MALAT1* expression was quantified by the RNA-FISH technique using aminoallyl-dUTP-Cy5-incorporated *MALAT1* probes prepared from asymmetric PCR amplification. The signals of aminoallyl-dUTP-Cy5–incorporated probes were used to visualize the hybridized probes. Using the Zeiss software module (Zen 2.6 Pro), the AMI was calculated by plotting the region of interest, as described previously [29].

Mfn2 Activity: The GTPase activity of Mfn2 was measured by immunoprecipitating Mfn2 from the cell homogenate, followed by quantifying the release of phosphate spectrophotometrically at 620 nm, as described previously [20].

*Mfn2* Promoter DNA Methylation: *Mfn2* promoter DNA methylation was determined by two independent techniques—quantifying 5mC levels and Dnmt1 binding at the *Mfn2* promoter. For 5mC quantification, the Methylamp™ Methylated DNA Capture Kit (Cat. No. P-1015-48, EPIGENTEK, Farmingdale, NY, USA) was used. Briefly, genomic DNA isolated from the cells using a DNeasy Blood & Tissue Kit (Cat. No. 69504, Qiagen, Valencia, CA, USA) was immunoprecipitated with 5mC or IgG antibodies, and 5mC levels were quantified by qRT-PCR using *Mfn2* promoter-specific primers. The qPCR values in each sample were normalized to the input sample controls by the 2^−ΔΔCt^ method [14].

*Mfn2* promoter DNA methylation was confirmed by quantifying Dnmt1 binding at the *Mfn2* promoter using the Chromatin Immunoprecipitation (ChIP) technique. Cells crosslinked with 1% formalin in PBS for 15 min and neutralized with 0.13 M glycine for 5 min were washed with PBS. Using protein-A agarose/salmon sperm DNA slurry, the protein extract was precleared and then immunoprecipitated with the Dnmt1 antibody. The antibody–protein-DNA complex was pulled down by protein A/G PLUS-Agarose beads and washed with low-salt and high-salt buffers, followed by a lithium chloride buffer. This was followed by washing twice with a Tris-EDTA buffer and reverse cross-linking. The complex was digested with proteinase K, and DNA was isolated by the phenol-chloroform-isoamyl alcohol-based method; qRT-PCR was performed using *Mfn2* promoter primers. The products were analyzed on 2% agarose gels to confirm the specificity of the ChIP assay. Each assay included input DNA as an internal control and IgG as the antibody control [14].

Chromatin Isolation by RNA Purification: To determine the binding of *MALAT1* at *Mfn2*, a chromatin extract was prepared from the cells crosslinked with 1% PFA, and the sheared chromatin extract was used for ChIRP, as described previously [29]. RNA immunoprecipitation-associated DNA fragments were quantified by qRT-PCR using primers for the *Mfn2* promoter region.

RNA Immunoprecipitation (RIP): The binding of *MALAT1* at Dnmt1 was investigated by the RIP technique; briefly, 1% formalin crosslinked cells were lysed and sheared by sonication. The clear lysate was incubated with 3 μg of Dnmt1 antibodies (Cat. No. ab13537, Abcam) or IgG (control antibody), and the complex was collected using protein A/G PLUS-Agarose beads. After washing the beads with a wash buffer, RNA was extracted using TRIzol, and cDNA was synthesized to quantify *MALAT1* transcripts by qRT-PCR [29,64].

Mitochondrial Fragmentation: Live cell imaging was performed on cells grown on coverslips and incubated with 200 nM MitoTracker green FM (Cat. No. M7514, Thermo Fisher Scientific, Waltham, MA, USA) for 15 min at 37 °C in a CO_2_ incubator. After washing the cells with PBS, they were imaged under a Zeiss ApoTome fluorescence microscope using a 63X objective. The mitochondrial outlines in the images were determined by ImageJ software (ImageJ, National Institutes of Health, Bethesda, MD, USA) [14,20]. The acquired mitochondrial images were converted to 8-bit images, and the aspect ratio (the ratio of the major axis and minor axis lengths) and form factor (4π.area/perimeter^2^) were calculated [42,43].

Electron Microscopy: Cells were fixed in a 0.1 M cacodylate buffer containing 2% paraformaldehyde–2% glutaraldehyde for two hours, and after washing them with the 0.1 M cacodylate buffer, they were incubated in 0.1 M cacodylate supplemented with 2% osmium and 3% potassium ferrocyanide for one hour on ice. Cells were stained with a 1% thiocarbohydrazide solution and incubated with 2% osmium at room temperature for 40 min, followed by 1% uranyl acetate at 4 °C overnight. After incubating the cells with Walton’s lead solution for 30 min at 60 °C, they were dehydrated using 50–100% ethanol and embedded in the resin for preparing ultrathin (70–85 nm) sections using Leica ARTOS 3D ultramicrotome (Leica, Teaneck, NJ, USA). The sections were placed on glow discharge-treated silicon wafers and imaged using a Zeiss Gemini300 scanning electron microscope (Carl Zeiss, Inc., Baden-Württemberg, Germany). The mitochondrial morphology and structure of the cristae were visualized using a backscatter detector [65]. The length of the mitochondria was quantified using ImageJ software with a size scale bar, and mitochondria were selected using the freehand line tool.

Oxygen Consumption Rate: OCR was determined using the Seahorse XF Cell Mito Stress Test Kit (Cat. No. 103015-100, Agilent Technologies, Santa Clara, CA, USA) according to the manufacturer’s protocol by injecting 1.5 µM oligomycin, 2.0 µM FCCP, and 0.5 µM rotenone/antimycin A in the ports A, B, and C, respectively. The data was collected and analyzed using the Wave software (10.3.1Correct, Agilent Technologies) [53].

Mitochondrial Membrane Potential: The membrane potential was determined using mitochondrial binding dye, JC-1 (Cat. No. MP03168, Molecular Probes, Carlsbad, CA, USA), as reported previously [20,30]. The ratio of J-aggregates (red fluorescence) to J-monomers (green fluorescence) was calculated.

Statistical Analysis: Statistical analyses were performed using GraphPad Prism 8 (San Diego, CA, USA), and the results are presented as mean ± SD. Significance of variance was determined using one-way ANOVA, and a *p* value < 0.05 was considered statistically significant.

## Figures and Tables

**Table 1:** Primer Sequence.

Primer	Sequences
*MALAT1*	Fwd-GCCATTCCAGGTGGTGGTATTTAG
	Rev-GCAGATTCTGTGTTATGCCTGGTTAG
*Mfn2*	Fwd-ATGCAGACGGAAAAGCACTT
	Rev-ACAACGCTCCATGTGCTGCC
*Mfn2* promoter	Fwd-TGCCCGATGAGTCACTTCAC
	Rev-CAAGGGGCGAAAAACCAAGG
*β-Actin*	Fwd-AGCCTCGCCTTTGCCGATCCG
	Rev-TCTCTTGCTCTGGGCCTCGTCG

## Data Availability

R.A.K. is the guarantor of this work and, as such, has full access to all the data in the study and takes responsibility for the integrity of the data and the accuracy of the data analysis.
